# Gut microbiota modulates lung gene expression and metabolism to aid SD rats in adapting to low-pressure hypoxia

**DOI:** 10.1128/spectrum.00045-25

**Published:** 2025-05-06

**Authors:** Zheng Chen, Shatuo Chai, Yuxia Ding, Kaiyue Pang, Tanqin Dong, Dongwen Dai, Jianmei Wang, Shuxiang Wang, Shujie Liu

**Affiliations:** 1 Qinghai Academy of Animal Husbandry and Veterinary Sciences in Qinghai University 207475, Xining, China; 2 College of Animal Science and Technology, China Agricultural University 462230 , Beijing, China; University of Nebraska-Lincoln, Lincoln, Nebraska, USA

**Keywords:** hypoxic pulmonary hypertension, gut microbiota, SCFAs, hypoxia adaptation, inflammation

## Abstract

Hypoxia has long posed a serious threat to the health of both animals and humans, causing respiratory acidosis, metabolic disorders, systemic inflammation, oxidative stress damage, and other issues, thereby endangering life and limiting development in high-altitude areas. Gut microbiota plays a crucial role in life activities and hypoxia adaptation. We transplanted the gut microbiota from small mammals, plateau zokors (*Myospalax baileyi*), from the Qinghai–Tibetan plateau (3,500 m) to Sprague–Dawley (SD) rats housed in a hypobaric chamber (equivalent to 6,000 m altitude) for 30 days. The results showed that microbiota transplantation significantly reshaped the gut microbiota structure of the rats, notably increasing the abundance of short-chain fatty acid-producing bacteria Lachnospiraceae and Prevotellaceae, alleviating hypoxia and acidosis, reducing pulmonary hypertension and right ventricular hypertrophy, increasing the production of anti-inflammatory substances like indole-3-lactic acid, and reducing the generation of pro-inflammatory substances, such as histamine and uric acid. It also decreased the expression of inflammatory genes like lgE, TNFα, and IFN-γ in the lung. Fecal microbiota transplantation from plateau-specific species to low-altitude SD rats effectively altered metabolism, changed gene expression, decreased pulmonary artery pressure, and enhanced plateau adaptability. This study demonstrates the potential effectiveness of treating hypoxic pulmonary hypertension through microbiota transplantation and offers insights into improving hypoxia adaptation.

**IMPORTANCE:**

We report the beneficial effects of FMT on respiratory capacity, lung metabolism, and lung gene expression in SD rats under hypoxic conditions. We revealed the inhibitory effects of gut microbiota on lung mast cells and histamine expression under hypoxic conditions. The study demonstrated the potential effectiveness of treating HPH through FMT and offers insights into improving hypoxia adaptation.

## INTRODUCTION

Currently, high-altitude regions (above 1,500 m) account for 30% of the world’s total land area, with over 200 million people living at altitudes exceeding 2,000 m (1). Additionally, some individuals need to travel to high-altitude areas for work, living, or other reasons. As a result, they must constantly face the challenges posed by the unique characteristics of high-altitude regions, such as low atmospheric pressure, reduced oxygen levels, and intense radiation (2, 3).

Moreover, the harsh high-altitude environment significantly impacts animal health as well. For instance, in practical livestock production on the Tibetan Plateau, high-yield Holstein dairy cows at an altitude of 2,500 m produce an average of only 20 kg of milk per day during their lactation peak, representing a nearly 50% reduction compared to their healthy state. At an altitude of 3,500 m, their physical condition deteriorates sharply, with many cows experiencing loss of appetite, lethargy, edema, pulmonary hypertension, and even death. Therefore, finding a solution to effectively address high-altitude damage is urgent.

Chronic exposure to hypoxic environments leads to several adverse physiological manifestations, including slow growth, increased blood viscosity, an increase in inflammatory cells, and hypoxic pulmonary hypertension (HPH) (4). HPH is particularly notable as one of the most formidable and life-threatening cardiovascular disorders (5). It is characterized by elevated pulmonary arterial pressure, increased pulmonary vascular resistance, and restructuring of the pulmonary vasculature (6). As the disease progresses, abnormalities in cardiac morphology and hemodynamics lead to right ventricular remodeling, ultimately culminating in right ventricular failure (7). Research has shown that gut microbiota plays a wide range of roles and is often referred to as the body’s “second brain” (8). Generally, hypoxia is accompanied by dysbiosis of the intestinal flora, which promotes intestinal mucus barrier dysfunction and systemic inflammation (9, 10). This condition leads to an increase in Desulfovibrionaceae and a decrease in Lachnospiraceae (11, 12). Lachnospiraceae, a butyrate-producing short-chain fatty acid (SCFA) bacterium, is crucial for maintaining the intestinal barrier and closely related to host life activities (13, 14).

Studies have shown that modifying the gut microbiota can alleviate hypoxic damage. By intervening in the gut microbiota, it is possible to improve the metabolism of SCFAs, bile acids, amino acids, neurotransmitters, and free fatty acids (15, 16). However, the effects and regulatory mechanisms of gut microbiota on respiratory capacity, lung metabolism, and lung gene expression under hypoxic conditions remain largely unexplored. Further studies are needed to elucidate these aspects, which will deepen our understanding of the relationship between gut microbiota and hypoxia adaptation and establish the interaction mechanisms of the gut–lung axis (17, 18). In light of this, we aim to transplant Sprague–Dawley (SD) rats with the gut microbiota of a plateau-specific species to allow us to explore the effects of gut microbiota on pulmonary arterial pressure, lung inflammatory compounds, and gene expression in SD rats exposed to low pressure and hypoxia.

The gut microbiota donor considered in this study focused on plateau zokors, small mammals endemic to the Qinghai–Tibet Plateau (2,500–5,000 m). These animals are well adapted to the alpine, anoxic, and resource-scarce environment of the plateau (19). Diet, environment, and host species all influence the composition of the animal gut microbiota. Differences in the gut microbiome composition lead to variations in functions, physiology, and adaptations (20). Studies have shown that the gut microbiota of plateau zokors is rich in species related to energy metabolism and SCFA production, such as Lachnospiraceae (21). This composition enables them to thrive in extreme climatic conditions on the plateau (22).

We hypothesized that transplantation of the gut microbiota from adaptive species specific to the Tibetan Plateau to SD rats at low altitudes could alter their gut microbiome, lung metabolism, cure or alleviate pulmonary hypertension, and improve their high-altitude adaptation. The results showed that fecal microbiota transplantation (FMT) significantly increased the abundance of short-chain fatty acid-producing bacteria in the rats' guts, regulated lung metabolism and gene expression, and attenuated hypoxia-induced pulmonary hypertension and right ventricular hypertrophy, enhancing their hypoxia adaptation.

## MATERIALS AND METHODS

### Animals and study design

Twenty-four 3-week-old male SD rats (weighing 55 ± 11 g) from Beijing Vital River Co., Ltd., China were randomly assigned to three groups: HAMG, HA, and H, with eight rats in each group. All rats were raised in homogeneous conditions in Xining, Qinghai (2,000 m) with free access to food and water under natural conditions. The body weight of all rats was recorded throughout the study. The rats in the HAMG group received antibiotic treatment, followed by transplantation of microbiota from plateau zokors, and were then raised under low pressure and hypoxic conditions. The rats in the HA group received antibiotic treatment without microbiota transplantation and were then raised under low pressure and hypoxic conditions. The rats in the H group received 10% PBS treatment and were then raised under low pressure and hypoxic conditions. The HA and H groups were employed as control groups, and the HAMG group was used as the experimental group.

Then, the HAMG and HA groups received a combination of vancomycin (0.5 g/L), ampicillin (1 g/L), neomycin (1 g/L), and metronidazole (1 g/L) *ad libitum* in their drinking water for 1 week. Subsequently, the HAMG and HA groups were continuously administered an antibiotic mixture solution (0.5 mL/50 g body weight, hereinafter the same) via gavage for 3 days. The purpose of the antibiotic treatment was to eliminate the host’s native microbiota, thereby preparing pseudo-germ-free rats (23–25). The H group, on the contrary, was given free access to drinking water for 1 week, followed by gavage with 10% PBS for 3 days.

Plateau zokors were initially live-trapped at an altitude of 3,000–3,500 m in Qinghai Province (*n* = 5). Three days later, an additional five zokors were live-trapped. Every day, fresh plateau zokor feces were collected, dissolved in PBS at a ratio of 1:10 (100 mg/1 mL), and mixed using a sterile stainless steel homogenizer (Waring 7011HS, Montreal, Canada). The mixture was then centrifuged at 600 *g* for 10 min (26). The rats in the HAMG group were given the supernatant by oral gavage right away.

After the antibiotic treatment and a 24 h withdrawal period, the HAMG group received fecal supernatant via gavage once daily for 7 days. The HA and H groups received 10% PBS via gavage daily for 7 days (27). Following the gavage treatments, after being kept at low altitude for 2 weeks, all rats were transferred to a low-pressure chamber (DYC-300, Feng Lei Co., Ltd., Guizhou, China) to simulate a hypoxic environment with an altitude of 6,000 m and an oxygen concentration of 9.1%. To reinforce the effect of microbiota transplantation, after entering the chamber, the rats in the HAMG group underwent gavage administration again for three consecutive days once daily. Daily cleaning and data recording of body weight and food intake were conducted. The rats were kept in this environment for a month, after which all rats were euthanized with urethane.

### Hemodynamics measurements

After 30 days of low pressure and hypoxic exposure, all rats were anesthetized with urethane (1.0 g/kg). The mean pulmonary artery pressure (mPAP) was measured using a right heart catheterization method (BL-420, Tai Meng Technology Co., Ltd., Chengdu, China). Following the measurements, the rats were immediately euthanized, and blood was collected from the abdominal aorta. The right and left ventricles plus ventricular septum (LV + S) were separated, washed to remove blood, weighed, and recorded. The right ventricular hypertrophy index (RVHI) was calculated using the formula RVHI = RV / (LV + S) (28).

### Blood gas analysis

After collecting arterial blood, it was sent to the First People Hospital of Qinghai for blood gas analysis using a GEM3000 analyzer (Beckman Coulter, USA). The analysis measured pH, partial pressure of carbon dioxide (PCO_2_), partial pressure of oxygen (PO_2_), base excess in the extracellular fluid (BEecf), concentration of bicarbonate (HCO_3_), and oxygen saturation (SO_2_).

### Analysis and sequencing of gut microbiota

Genomic DNA from fecal samples was extracted using the e.zn.a DNA Kit (Omega Bio-Tek, Norcross, GA, USA). The purity and concentration of the DNA were then monitored, and its quality was checked using 1% agarose gel electrophoresis. The V3–V4 region of the 16S rRNA gene was amplified by polymerase chain reaction. PCR products were extracted from 2% agarose gel and purified using the AxyPrep DNA Gel Extraction Kit (AxyPrep Biosciences, Union City, CA, USA). Finally, the high-quality amplicons were sequenced on the Illumina MiSeq PE300 platform (San Diego, CA, USA). Sequences with over 97% similarity were clustered into operational taxonomic units (OTUs), and statistical analyses were performed on OTU clustering, species taxonomy, diversity indices, and community structure.

### Analysis of SCFA

The SCFA content in the fecal samples was determined using a Shimadzu GC-2014 Gas Chromatograph. After thawing the fecal samples, approximately 1 g was dissolved in 10 mL of MilliQ water and centrifuged at 3,000 r/min for 10 min. Then, 2 mL of the supernatant was transferred to a 5 mL centrifuge tube, and 400 µL of metaphosphoric acid solution was added. The mixture was thoroughly mixed and placed in an ice-water bath for 30 min. Subsequently, it was centrifuged at 12,000 r/min for 10 min at 4°C, and the supernatant was collected for analysis.

### Lung metabolism

The lung tissue was ground, and the supernatant was collected for analysis using liquid chromatography–mass spectrometry. The resulting data (.raw files) were imported into CD 3.1 software for processing, where each metabolite was screened based on retention time and mass-to-charge ratio. The raw quantitative results were standardized to obtain the identification and the relative quantification of metabolites. Subsequently, differential analysis was performed on the data to obtain the variable importance in the projection (VIP) value for each metabolite. The statistical significance (*P* value) of metabolites between the two groups was calculated using a *t*-test, and the fold change between the groups was determined. The criteria for selecting differential metabolites were VIP > 1.0, FC > 1.5, and *P* value < 0.05. Data processing was conducted using the Linux Operating System (CentOS version 6.6), R, and Python software

### Lung RNA-Seq analysis

Lung tissue samples were analyzed in NOVOGENE Company Limited (Beijing, China). Briefly, RNA integrity was assessed using the RNA Nano 6000 Assay Kit, and RNA was purified using poly-T oligo-attached magnetic beads. The library products were prepared for sequencing in Illumina NovaSeq 6000. The fragments per kilobase of transcript per million mapped fragments for each gene were calculated using the gene length and corresponding read counts. Differential expression analysis was performed using DESeq2R software (version 1.20.0). The Benjamini–Hochberg method was applied for multiple test correction (false discovery rate < 0.05). Genes with |log2(FoldChange)| > 0 and *P* value < 0.05 were assigned as differentially expressed. Data were then analyzed by Kyoto Encyclopedia of Genes and Genomes (KEGG) enrichment.

### Statistical analysis

Weight, mPAP, and other data were analyzed using SAS 9.4 (SAS Institute, Inc., Cary, NC, USA) with a one-way analysis of variance (ANOVA). The means ± standard deviation were used to display the values. At *P* < 0.05, statistical significance was established.

## RESULTS

### Gut microbiota improves growth, development, and pulmonary hypertension

**Fig 1:**
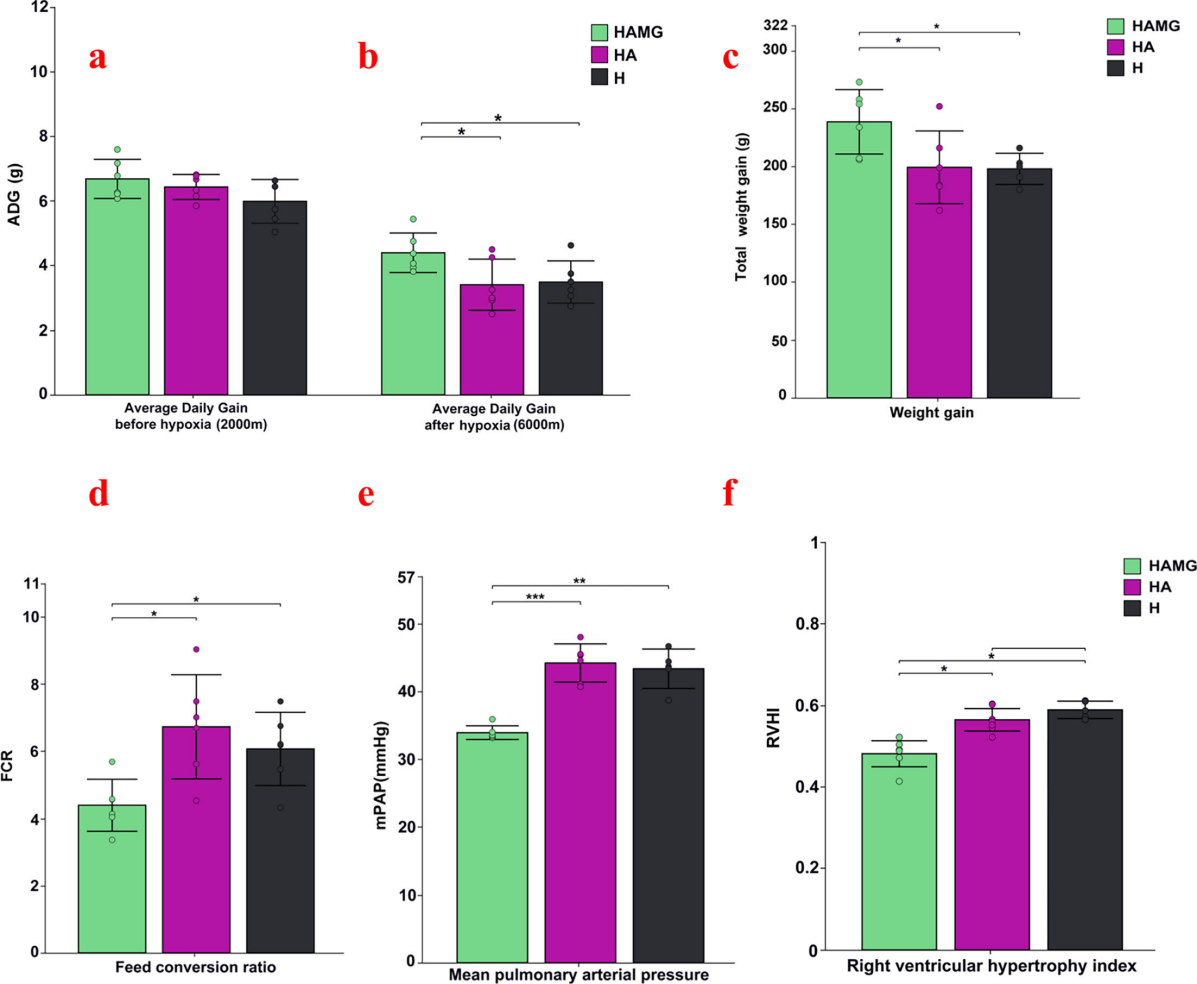
Fecal microbiota transplantation modulates weight gain and alleviates pulmonary arterial hypertension. (**a**) Average daily gain before hypoxia (altitude: 2,000 m). (**b**) Average daily gain after hypoxia (altitude; 6,000 m). (**c**) Total weight gain. (**d**) Feed conversion ratio after hypoxia (altitude: 6,000 m). (**e**) Mean pulmonary arterial pressure across all groups. (**f**) Right ventricular hypertrophy index across all groups. *P ≤ 0.05, **P ≤ 0.01, and ***P ≤ 0.001. No asterisk indicates no significant differences.

As a result of chronic hypoxia, attrition was observed across all SD rat groups during the experimental period. The final numbers were six rats in the HAMG group, six rats in the HA group, and five rats in the H group. As shown in Fig. 1, the average daily gain (ADG) before hypoxia in the three groups was HAMG (6.68 ± 0.27 g) > HA (6.43 ± 0.49 g) > H (6.01 ± 0.36 g), with no significant differences (*P* > 0.05) (Fig. 1a). After hypoxia, the ADG in the rats was HAMG (4.39 ± 0.25 g) > H (3.47 ± 0.37 g) > HA (3.40 ± 0.19 g). The ADG in the HAMG group was significantly higher than in the control groups (*P* < 0.05), with no significant difference between HA and H (*P* > 0.05) (Fig. 1b). The total weight gain of the HAMG, HA, and H was (238.66 ± 8.9 g) > (199.33 ± 6.3 g) > (198.10 ± 5.8 g), with the HAMG group being significantly higher than the two control groups (Fig. 1c). The feed conversion ratio in the three groups was HAMG (4.39 ± 0.58) < H (6.03 ± 0.84) < HA (6.53 ± 1.12) (*P* < 0.05) (Fig. 1d). The mPAP in HAMG, HA, and H were 34.01 ± 0.31, 44.21 ± 1.22, and 43.45 ± 1.30 mmHg, respectively. Compared to the control groups, the mPAP of the HAMG group was noticeably lower. There was no statistically significant difference in mPAP between the two control groups (Fig. 1e). The trend in RVHI was consistent with that of mPAP (Fig. 1f).

### FMT affected blood gas values

**Fig 2:**
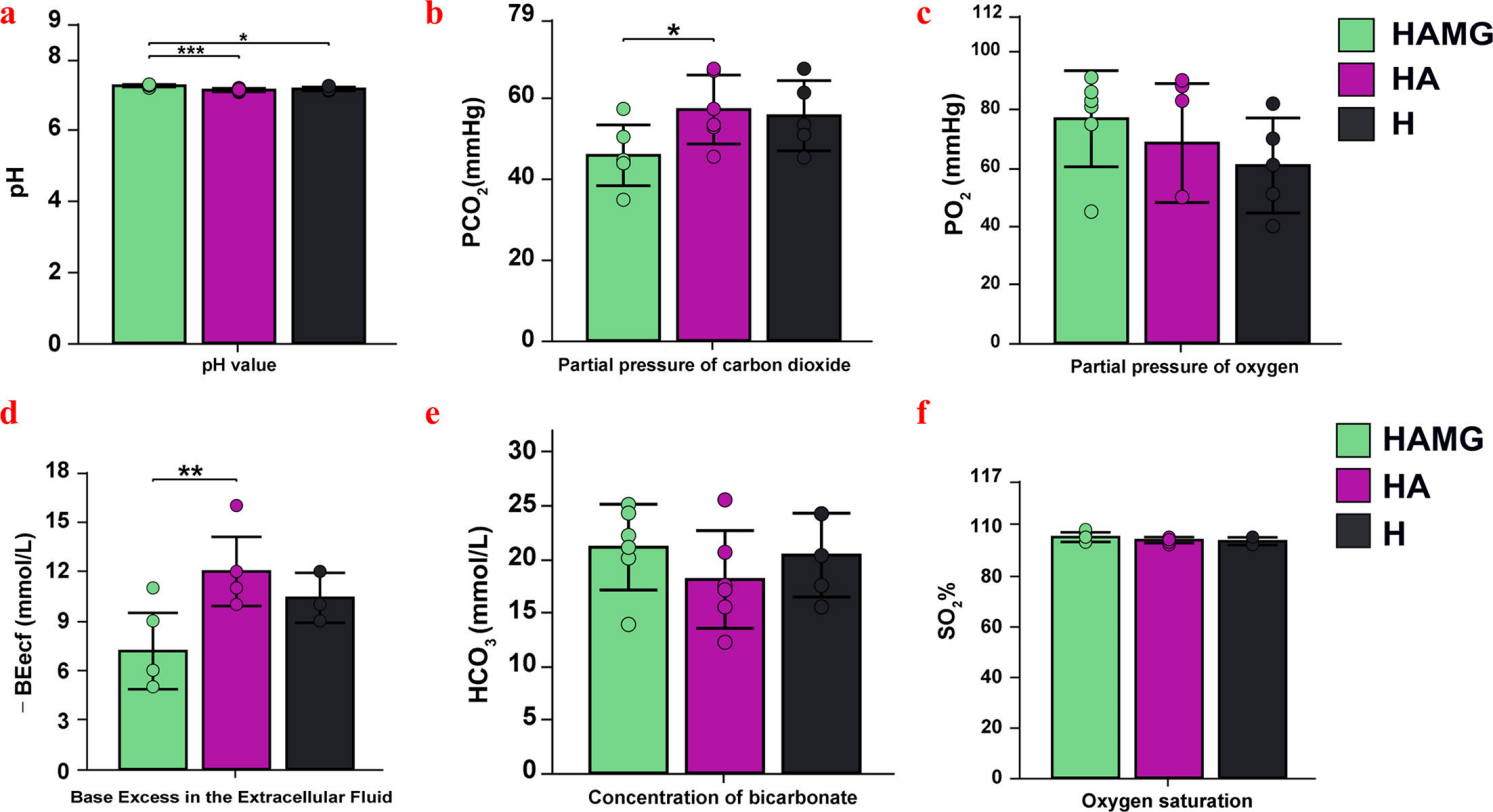
Impact of fecal microbiota transplantation on blood gas parameters in rats. (**a**) Blood pH value across all groups. (**b**) Blood PCO_2_ across all groups. (**c**) Blood PO_2_ across all groups. (**d**) Blood BEecf across all groups. (**e**) Blood HCO_3_ across all groups. (**f**) Blood SO_2_% across all groups. *P ≤ 0.05, **P ≤ 0.01, and ***P ≤ 0.001. No asterisk indicates no significant differences.

As shown in Fig. 2, the pH values for the groups were HAMG (7.26 ± 0.04) > H (7.17 ± 0.05) > HA (7.14 ± 0.05) (*P* < 0.05). The pH in the HAMG group was significantly higher than in the two control groups, with no significant difference between the controls (*P* > 0.05) (Fig. 2a). The PCO_2_ values in the HAMG, HA, and H groups were 45.65 ± 7.49 < 56.95 ± 8.50 < 55.42 ± 8.42 mmHg, respectively. The PCO_2_ in the HAMG group was significantly lower than in the HA group (*P* < 0.05), with no significant differences among the other groups (*P* > 0.05) (Fig. 2b). The PO_2_ values were HAMG (76.83 ± 16.67 mmHg) > HA (68.5 ± 20.89 mmHg) > H (60.81 ± 16.30 mmHg), with no significant differences among the groups (*P* > 0.05) (Fig. 2c). The BEecf values were HAMG (−7.17 ± 2.32 mmol/L) > H (−10.4 ± 1.51 mmol/L) > HA (−12.00 ± 2.09 mmol/L) (*P* < 0.05) (Fig. 2d). The HCO_3_ values in the HAMG, HA, and H groups were 21.31 ± 4.01, 18.07 ± 4.56, and 20.34 ± 3.91 mmol/L, respectively, with no significant differences among the groups (*P* > 0.05) (Fig. 2e). The SO_2_% values were HAMG (95.00 ± 1.90%), HA (93.84 ± 1.17%), and H (93.40 ± 1.57%), with no significant differences among the groups (*P* > 0.05) (Fig. 2f).

### FMT contributed to different composition of gut microbiota

**Fig 3:**
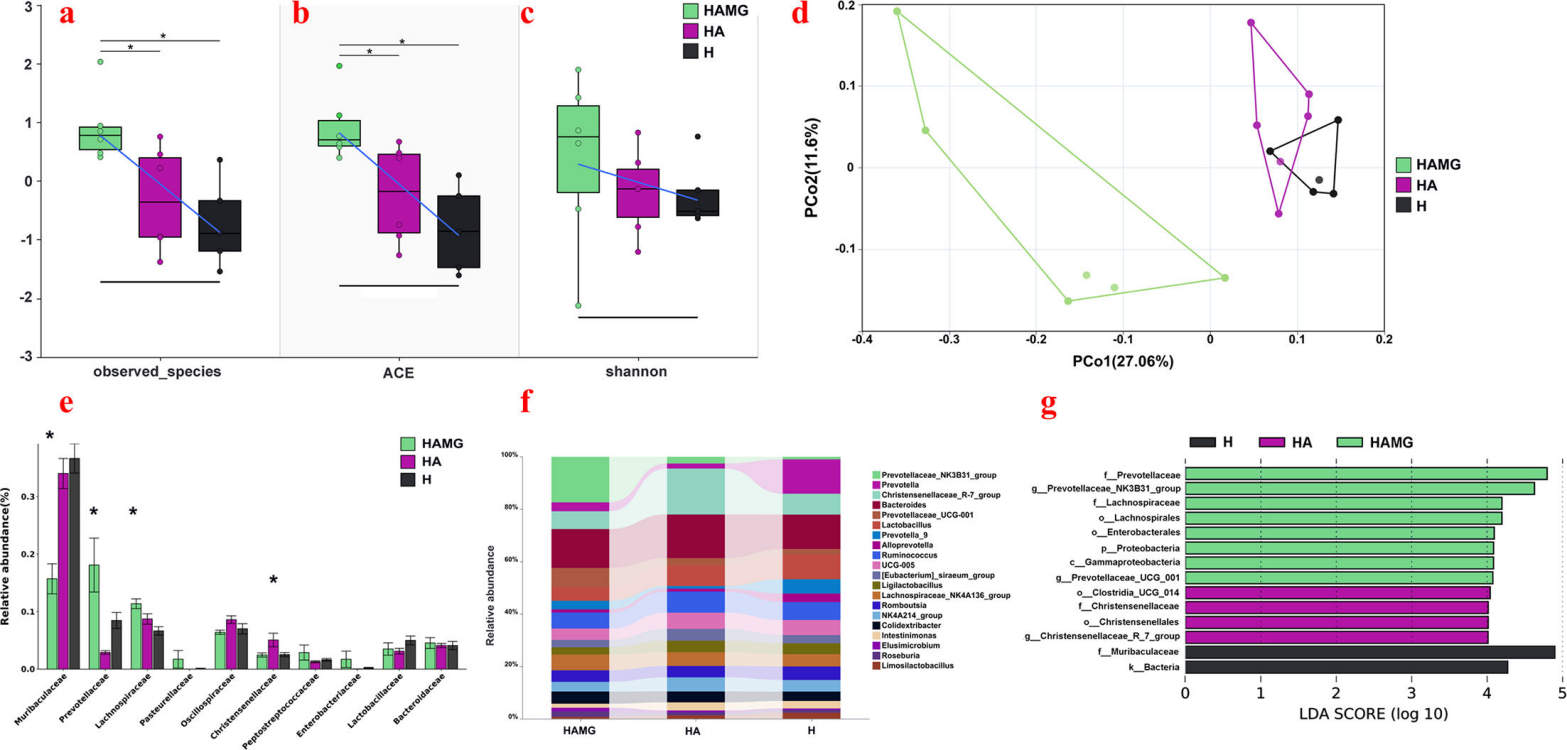
Comparison of the gut microbiome among the HAMG, HA, and H groups. (**a**) Observed species of α-diversity across all groups. (**b**) ACE index of α-diversity across all groups. (**c**) Shannon index of α-diversity across all groups. (**d**) PCoA analysis based on the Unweighted-UniFrac distance. (**e**) Bar chart of differences at the top 10 family level. * indicates significant differences compared to the other two groups. (**f**) Top 20 genera-level relative abundance. (**g**) Linear discriminant analysis effect size was used to identify biomarkers in each group, and the LDA score was 4.0.

A 16S rRNA analysis on fecal samples was performed to explore the microbial community composition among the groups. The alpha diversity analysis showed that Chao1 was significantly higher in the HAMG (1,038.33 ± 25.44) group than in the HA (986.17 ± 38.17) and H groups (968.40 ± 32.29) (*P* < 0.05), with no significant differences among the other groups (*P* > 0.05) (Fig. 3a). The ACE index was HAMG (1084 ± 23.88), HA (1036 ± 35.12), and H (1012 ± 31.89), with no significant differences among the groups (*P* > 0.05) (Fig. 3b). The Shannon index in the HAMG, HA, and H groups was 7.50 ± 0.39, 7.36 ± 0.20, and 7.34 ± 0.16, respectively, with no significant differences among the groups (*P* > 0.05) (Fig. 3c). The principal coordinate analysis based on unweighted UniFrac distances showed significant differences in microbial community composition among the groups (*P* < 0.05) (Fig. 3d). The permutational multivariate analysis of variance with 999 permutations yielded the following *P* values: HAMG vs. HA, *P* value = 0.001; HAMG vs. H, *P* value = 0.002; and HA vs. H, *P* value = 0.003.

At the phylum level, according to Fig. S1, Elusimicrobia, Euryarchaeota, and Proteobacteria were enriched in the HAMG group. Verrucomicrobiota and Firmicutes were enriched in the HA group. Bacteroidota was enriched in the H group. At the family level, the predominant bacteria in the control groups were Muribaculaceae, which were significantly higher than in the HAMG group (*P* < 0.05). Lachnospiraceae and Prevotellaceae were enriched in the HAMG group (*P* < 0.05). Christensenellaceae was enriched in the HA group (*P* < 0.05) (Fig. 3e). Twenty genera are shown in Fig. 3f and Fig. S2. *Prevotellaceae_UCG-001*, *Prevotellaceae_NK3B31_group*, and *Lachnospiraceae_NK4A136_group* were enriched in the HAMG group. *Christensenellaceae_R-7_group*, *[Eubacterium]_siraeum_group*, *Ruminococcus*, and *NK4A214_group* were enriched in the HA group. *Prevotella*, *Alloprevotella*, and *Limosilactobacillus* were enriched in the H group. We used a linear discriminant analysis effect size analysis to further identify biomarkers with a linear discriminant analysis (LDA) score greater than 4.0 (Fig. 3g). The results showed that Prevotellaceae, Lachnospiraceae, *Prevotellaceae_NK3B31_group*, and *Prevotellaceae_UCG_001* were enriched in the HAMG group. Christensenellaceae and *Christensenellaceae_R_7_group* were enriched in the HA group. Bacteria and Muribaculaceae were enriched in the H group.

### Different gut microbiota with different SCFAs

**Fig 4:**
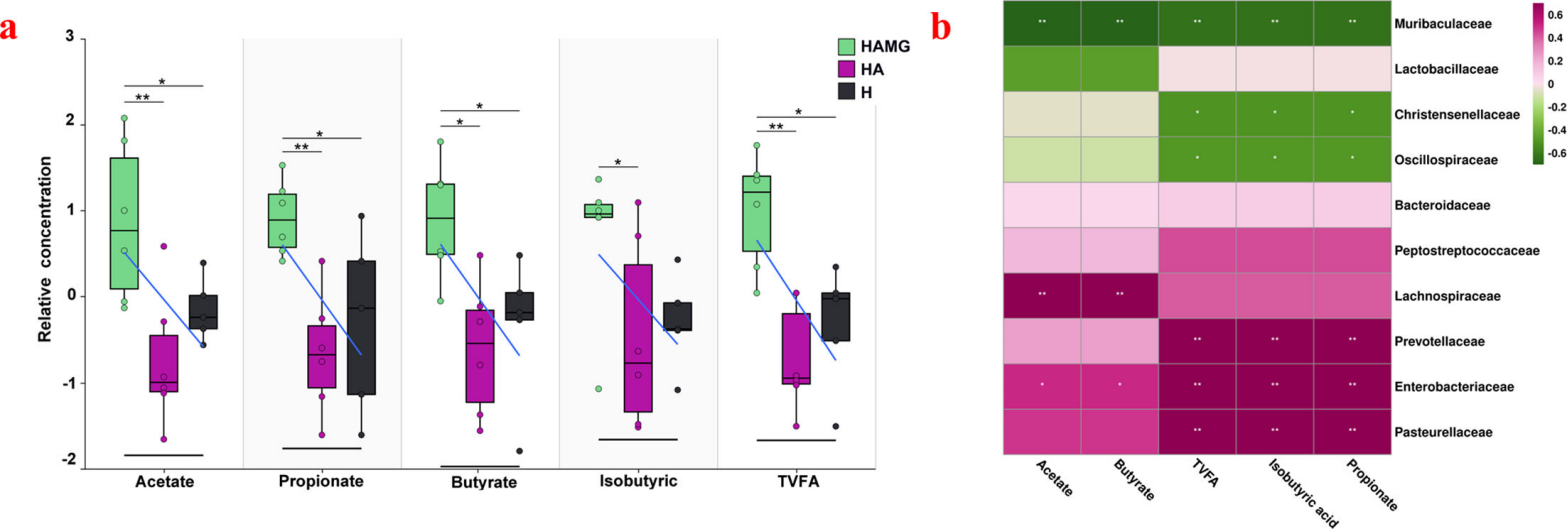
The gut microbiota modulates the metabolism of SCFAs. (**a**) Relative concentrations of SCFAs (acetate, propionate, butyrate, isobutyrate, and total volatile fatty acids) in fecal samples across all groups. *P ≤ 0.05, **P ≤ 0.01, and ***P ≤ 0.001. No asterisk indicates no significant differences. (**b**) Spearman correlation between the top 10 families and SCFAs. Red indicates a positive correlation, while green indicates a negative correlation. *P ≤ 0.05, **P ≤ 0.01, and ***P ≤ 0.001.

The gas chromatography analysis of SCFAs in fecal samples showed that the relative yields of acetate, propionate, butyrate, and total volatile fatty acids (TVFA) were significantly higher in the HAMG group (*P* < 0.05), with no significant differences between the HA and H groups (*P* > 0.05). The yield of isobutyric acid was significantly higher in the HAMG group compared to the HA group (*P* > 0.05), but there was neither a significant difference between the HAMG and H groups (*P* < 0.05) nor between the HA and H groups (*P* < 0.05) (Fig. 4a).

To confirm the impact of different bacterial families on SCFA production, we performed a Spearman correlation analysis. The results showed that Lachnospiraceae was significantly positively correlated with acetate and butyrate (*P* < 0.05). Prevotellaceae was significantly positively correlated with propionate, isobutyric acid, and TVFA (*P* < 0.05). Muribaculaceae was significantly negatively correlated with acetate, propionate, butyrate, isobutyric acid, and TVFA (*P* < 0.05). Christensenellaceae was significantly negatively correlated with propionate, isobutyric acid, and TVFA (*P* < 0.05) (Fig. 4b).

### Different gut microbiota with different lung metabolism

**Fig 5:**
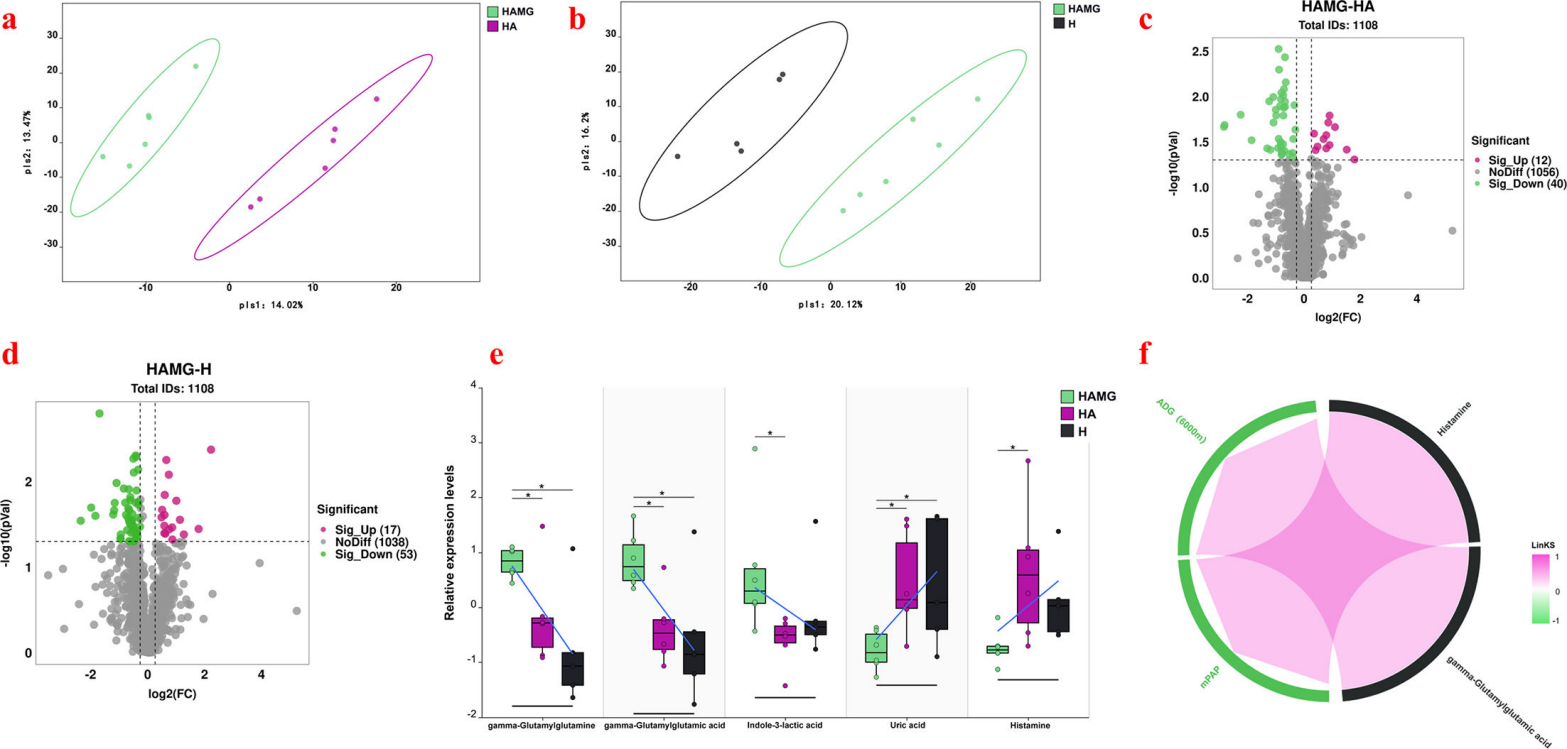
Effects of fecal microbiota transplantation on lung metabolism. (**a**) PLS-DA analysis of lung metabolites in the HAMG and HA groups. (**b**) PLS-DA analysis of lung metabolites in the HAMG and H groups. (**c**) Volcano plot of lung metabolites in the HAMG and HA groups. (**d**) Volcano plot of lung metabolites in the HAMG and H groups. (**e**) Relative expression levels of gamma-glutamylglutamine, gamma-glutamylglutamic acid, indole-3-lactic acid, uric acid, and histamine across all groups. *P ≤ 0.05, **P ≤ 0.01, and ***P ≤ 0.001. No asterisk indicates no significant differences. (**f**) Correlation analysis of gamma-glutamylglutamine, gamma-glutamylglutamic acid, indole-3-lactic acid, uacid, and histamine with average daily gain after hypoxia (6,000 m) and mPAP. The correlation chord diagram shows red chords representing positive correlations and green chords representing negative correlations. Each node represents a substance, with the node color indicating the group, meaning different colored nodes represent different groups.

We used liquid chromatography–tandem mass spectrometry for lung metabolomics analysis to explore metabolic differences in the lungs. The heatmap results showed that the HAMG group had significantly different metabolites compared to the HA and H groups (Fig. S3). The partial least squares discriminant analysis plot showed a clear separation between the HAMG and HA and H groups (Fig. 5a and b). Volcano plot analysis revealed a total of 1,108 metabolites detected in the lung tissue. Compared to the HA group, 12 metabolites were significantly upregulated, and 40 metabolites were significantly downregulated in the HAMG group (Fig. 5c). Compared to the H group, 17 metabolites were significantly upregulated, and 53 metabolites were significantly downregulated in the HAMG group (Fig. 5d).

Using the KEGG pathway analysis, we annotated the functions of metabolites. The differential metabolites between the HAMG and HA groups were enriched in pathways, such as vitamin digestion and absorption, vitamin B6 metabolism, tryptophan metabolism, taurine and hypotaurine metabolism, and insulin resistance (Fig. S4). The differential metabolites between the HAMG and H groups were enriched in pathways, such as protein digestion and absorption, vancomycin resistance, sulfur metabolism, and starch and sucrose metabolism (Fig. S5). Through a functional analysis of all metabolites, we identified five key metabolites. Gamma-glutamylglutamine, gamma-glutamylglutamic acid, and uric acid were significantly higher in the HAMG group compared to the two control groups (*P* < 0.05), with no significant differences between the control groups (*P* > 0.05). Indole-3-lactic acid and histamine were significantly higher in the HAMG group compared to the HA group (*P* < 0.05), with no significant differences among the other groups (*P* > 0.05) (Fig. 5e).

To determine the impact of different metabolites on weight gain and mPAP, we performed a Spearman correlation analysis. The results showed that histamine was significantly positively correlated with mPAP, and gamma-glutamylglutamic acid was significantly positively correlated with ADG (6000 m) (Fig. 5f).

### Gut microbiota affected the gene expression profiles of the lung

**Fig 6:**
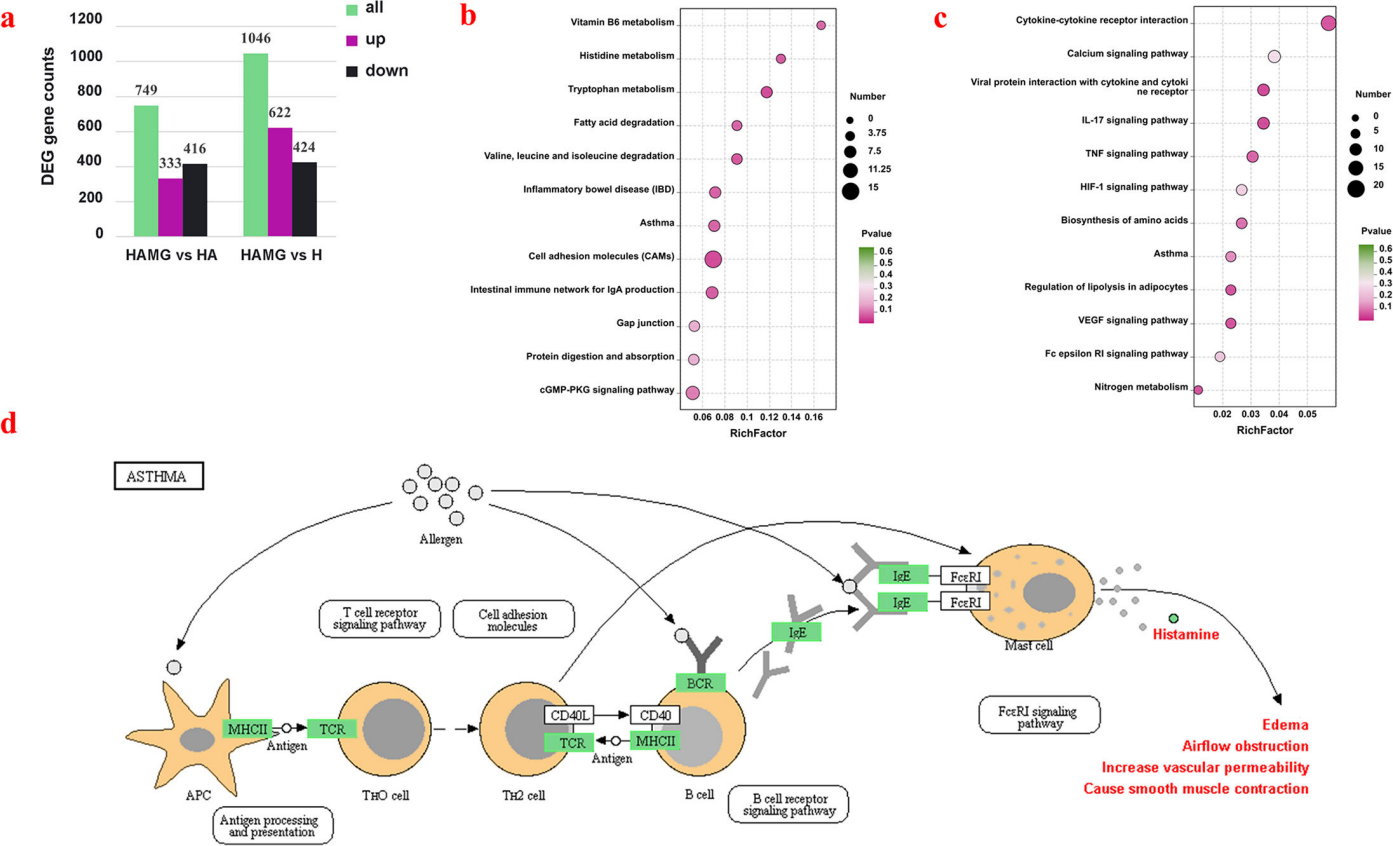
Fecal microbiota transplantation alters lung gene expression. (**a**) Bar chart of differential gene expression. (**b**) KEGG-enriched pathways of differential genes in HAMG vs. HA. (**c**) KEGG-enriched pathways of differential genes in HAMG vs. H. (**d**) Asthma pathway diagram. Genes marked in green indicate those downregulated in the HAMG group compared to the control groups.

To further investigate the effect of gut microbiota transplantation, gene expression profiles for the lung were quantified by an RNA-seq analysis. The co-expression Venn diagram shows that 15,231 genes were annotated in lung tissue, with 227 genes uniquely expressed in the HAMG group, 221 genes uniquely expressed in the HA group, and 250 genes uniquely expressed in the H group (Fig. S6). Comparing the differential gene expressions among the groups, a total of 333 genes were upregulated, while 416 genes were downregulated by HAMG compared to HA; 622 genes were upregulated, while 424 genes were downregulated by HAMG compared to H (Fig. 6a). In comparison to the H group, the HA group showed upregulation of 591 genes and downregulation of 486 genes (Fig. S7). The functions of the altered genes were determined by the KEGG pathway analysis. In the HAMG and H groups, the differentially expressed genes were primarily enriched in metabolic pathways, such as inflammatory bowel disease, cell adhesion molecules, tryptophan metabolism, intestinal immune network for IgA production, asthma, and gap junctions (Fig. 6b). In the HAMG and HA groups, the differentially expressed genes were mainly enriched in the IL-17 signaling pathway, VEGF signaling pathway, regulation of lipolysis in adipocytes, TNF signaling pathway, and biosynthesis of amino acids (Fig. 6c). In the HA and H groups, the differentially expressed genes were primarily enriched in lysosome, osteoclast differentiation, protein digestion and absorption, hematopoietic cell lineage, and phagosome (Fig. S8). Through a combined metabolomic analysis, we found a significant metabolic pathway, the asthma pathway. The pathway indicated that, compared to the control groups, the HAMG group exhibited significantly downregulated expressions of MHCII, TCR, BCR, and IgE, which in turn affected the histamine expression. This further influenced physiological functions, such as airflow obstruction, increased vascular permeability, and smooth muscle contraction (Fig. 6d).

**Fig 7:**
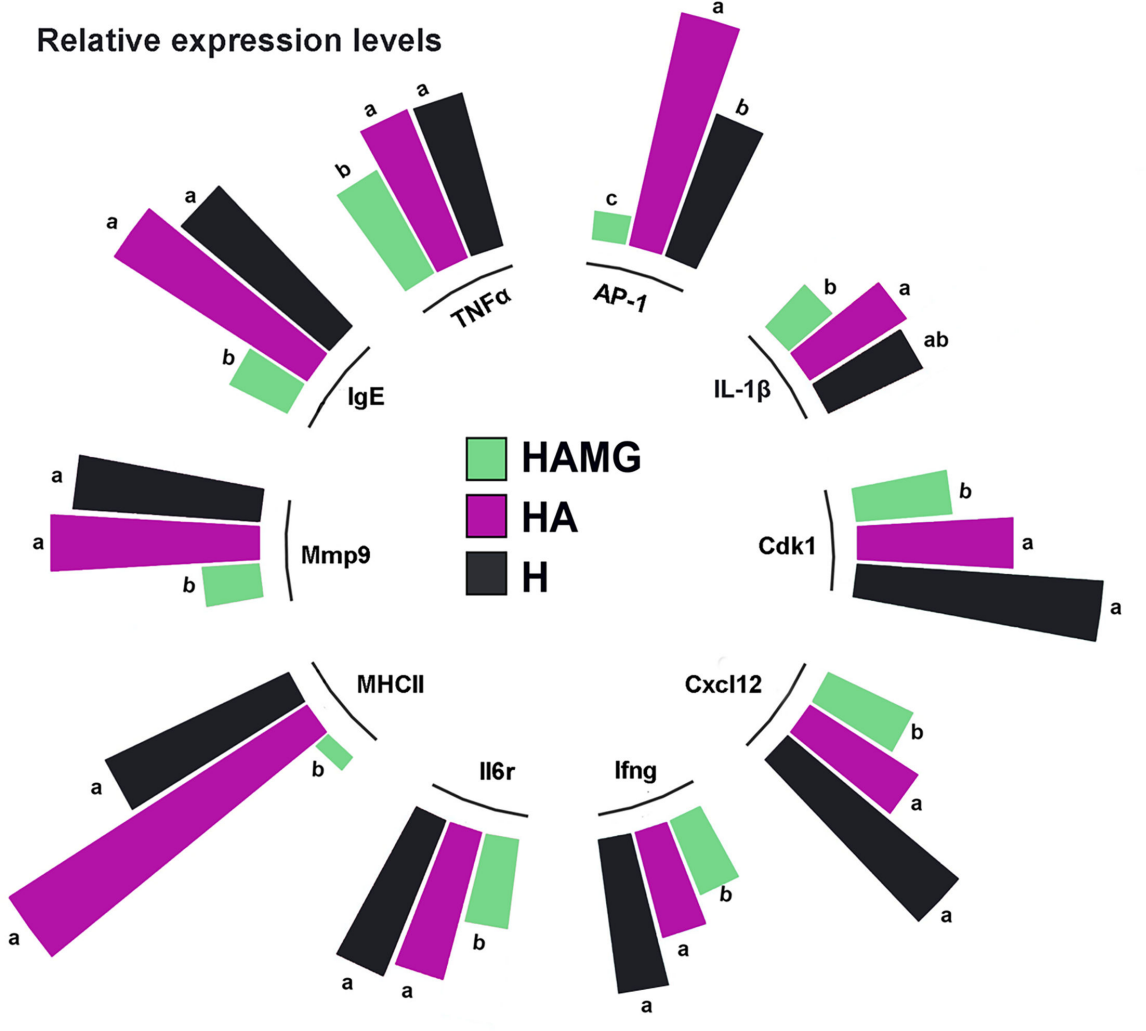
Relative expression of 10 key genes in HAMG, HA, and H. Genes with a *P* value < 0.05 and |log2(FoldChange)| > 0 were considered differentially expressed. Columns with different lowercase letter superscripts within the same gene are significantly different (*P* < 0.05).

Through further analysis of the differentially expressed genes, we found that the expression levels of AP-1, IL-1β, Cdk1, Cxcl12, Ifng (IFN-γ), Il6r, MHCII, Mmp9, IgE, and TNFα were significantly reduced in the HAMG group. These genes may be associated with inflammation and tissue remodeling in chronic low-pressure hypoxic environments (Fig. 7).

## DISCUSSION

The capacity of animals to adapt to the environment can be partially reflected in their growth and development rates (29). Hypoxia has been shown to reduce energy intake in animals, leading to weight loss (30). Studies have shown that rumen fluid transplantation from yaks on the Qinghai–Tibet Plateau improves growth performance, reshapes rumen microbiota, and enhances feed efficiency in yaks (31). In this study, FMT reshaped the gut microbial community in rats, and the transplantation group showed significantly improved growth and development compared to the control group, along with enhanced feed efficiency, reflecting an improvement in hypoxia adaptation. Additionally, the transplantation group exhibited significantly lower mPAP and RVHI than the control group, indicating that FMT significantly alleviated pulmonary vascular pressure and right ventricular remodeling. This suggests that the transplantation group had healthier lungs and a potentially better respiratory capacity. Blood gas values are crucial indicators of the body’s acid–base balance, pulmonary ventilation, and respiratory function (32, 33). Through blood gas analysis, we observed that all three groups exhibited not only respiratory acidosis but also severe metabolic acidosis, resulting in a mixed acid–base imbalance. However, compared to the control group, the transplantation group showed significant improvements in pH, PCO_2_, and BEecf values. This suggests that the gut microbiota alleviated respiratory distress in rats, enhanced respiratory function, reduced the severity of hypoxia, and improved their adaptability to high-altitude environments. These results demonstrated that reshaping the gut microbiota of rats through FMT significantly improved their health under high-altitude conditions.

Gut microbiota analysis revealed that the transplantation group showed a marked enrichment of Lachnospiraceae, Prevotellaceae, Prevotellaceae_UCG-001, Prevotellaceae_NK3B31_group, and Lachnospiraceae_NK4A136_group. Research has shown that these bacteria are significant producers of SCFAs, which are essential for maintaining gut health, regulating metabolism, and supporting overall growth and development in animals (34, 35). Lachnospiraceae and Prevotellaceae have various functions, including polysaccharide degradation, antioxidant stress, and anti-inflammatory effects, making them potential biomarkers of health (36, 37). Under hypoxic conditions, they help regulate oxidative stress, reduce inflammation, and protect the gut barrier, aiding the host in adapting to hypoxia. Studies have shown that Lachnospiraceae are beneficial for high altitude-related cardiac hypertrophy and pulmonary hypertension, as they help maintain low uric acid levels and enhance the host’s adaptation to high-altitude hypoxia (38). Notably, in this study, the lung uric acid levels in the transplantation group were significantly lower than those in the control group. *Prevotellaceae_UCG-001*, *Prevotellaceae_NK3B31_group*, and Lachnospiraceae_NK4A136_group, which belong to Lachnospiraceae and Prevotellaceae, not only control inflammation and regulate the host’s immune system but also improve the host’s growth performance (39–42). In this study, the transplantation group showed significantly higher growth levels and feed conversion rates compared to the control group. The analysis of SCFAs also indicates that the transplantation group exhibited a better metabolic performance, which is a crucial factor in enhancing the hypoxia adaptability of the rats. SCFAs play crucial roles in antioxidant stress, maintaining electrolyte balance, anti-inflammation, regulating gut microbiota balance, modulating immunity (43, 44), and controlling gene expression, all of which are vital to the body’s life activities (45, 46). Numerous studies have shown that SCFAs can alleviate hypoxia-induced pulmonary hypertension, pulmonary vascular remodeling, and inflammation (47, 48).

To further confirm the impact of gut microbiota on the lungs and establish the gut–lung axis connection, we explored lung metabolism and gene expression. It is evident that microbiota transplantation significantly altered lung metabolism and gene expression. In terms of metabolic pathways, the transplantation group showed significant changes in lipid metabolism, glucose metabolism, and amino acid metabolism compared to the control group. Investigating the functional relevance of differential metabolites, we found that histamine, uric acid, and indole-3-lactic acid may play key roles during prolonged hypoxia (49). Histamine is a potent inflammatory mediator that can cause pulmonary artery vasoconstriction and increase pulmonary vascular resistance (50, 51). Prolonged exposure to a hypoxic environment leads to increased histamine release from mast cells and other immune cells, exacerbating inflammatory responses, causing tissue damage, and worsening HPH (52). In both lung metabolism and transcriptome analyses, histamine-related metabolic pathways were annotated. The transplantation group showed a significant downregulation of histamine-related genes compared to the control group. For example, IgE antibodies play a crucial role in inflammatory responses and allergic reactions (53). Reducing IgE levels or inhibiting the FcεRI receptor can be an effective anti-inflammatory strategy (54). IgE binds to high-affinity surface receptors on mast cells, leading to the release of proinflammatory mediators, such as histamine (55, 56). Notably, studies have shown that SCFAs, particularly butyrate, can inhibit the IgE-mediated degranulation process of mast cells, regulate mast cell gene expression and activity, and reduce inflammatory responses (57). This is consistent with the findings of our study. Uric acid, a purine metabolite, is associated with inflammation, insulin resistance, and gout (58–60). Studies have shown that high uric acid levels are closely linked to tissue hypoxia and oxidative stress commonly observed in patients with pulmonary hypertension, which aggravates the development of HPH. In this study, the transplanted group showed a significant reduction in the lung tissue (61–63). Indole-3-lactic acid, a metabolite of tryptophan metabolism, has functions, such as reducing intestinal inflammation (64), combating oxidative stress, and protecting epithelial cells (65). It plays an important role in maintaining gut health and regulating the gut microbiota (66). Research indicated that individuals who follow a healthy Mediterranean diet experience an increase in the abundance of Lachnospiraceae in their feces, as well as an increase in the expression of indole-3-lactic acid (67). In this study, the abundance of Lachnospiraceae and the expression level of indole-3-lactic acid were significantly elevated in the transplantation group, suggesting that indole-3-lactic acid could be a potential therapeutic agent to mitigate the adverse effects of hypoxia on gut and lung health.

In terms of gene expression, further research revealed that the expression of inflammatory genes, such as IL-1β, Cdk1, Cxcl12 (68, 69), Ifng (IFN-γ), Il6r, Mmp9, and TNFα, was significantly reduced in the lungs of the transplantation 70–72). They have all been proven to be significant contributors to the development of pulmonary hypertension. For example, matrix metalloproteinases are enzymes that degrade extracellular matrix components. In the lung tissue of rats with pulmonary hypertension, Mmp9 expression is upregulated (73). TNFα is activated in hypoxic environments, inducing pulmonary vasoconstriction and playing a crucial role in the inflammatory response under hypoxic conditions, exacerbating HPH (74). Anti-inflammatory treatments targeting TNFα may offer a new approach for HPH therapy. The reduced expression of these inflammatory genes indicates significantly decreased lung inflammation in the transplantation group, resulting in healthier lungs. Overall, our study demonstrates the significant regulatory role of gut microbiota in pulmonary metabolism and gene expression under hypoxic conditions. FMT can improve pulmonary metabolism and reduce the expression of inflammatory genes and mediators. Diseases caused by chronic hypoxia are fundamentally metabolic disorders. Investigating HPH from the perspective of improving systemic metabolism in animals provides deeper insights into HPH. However, our research has only explored the role of SCFA-producing bacteria and SCFAs. Deeper connections and mechanisms require further research and elucidation.

### Conclusion

In summary, this study demonstrated that the gut microbiota plays a crucial role in reducing the development of HPH and enhancing hypoxia adaptation in rats. These effects may be due to the enrichment of short-chain fatty acid-producing bacteria, Lachnospiraceae and Prevotellaceae, thereby regulating lung metabolism and gene expression and alleviating pulmonary inflammation and oxidative stress. This study indicates the potential effectiveness of treating HPH through microbiota transplantation and offers insights into improving hypoxia adaptation.

## Data Availability

The 16s rRNA gene sequencing data have been uploaded to NCBI under accession number PRJNA894774. The transcriptomics and metabolomics data have been deposited in the China National GeneBank DataBase (CNGBdb) under accession number CNP0003641.

## SUPPLEMENTAL MATERIAL

Supplemental material spectrum.00045-25-s0001.docx Fig. S1 to S12.

ASM does not own the copyrights to Supplemental Material that may be linked to, or accessed through, an article. The authors have granted ASM a non-exclusive, world-wide license to publish the Supplemental Material files. Please contact the corresponding author directly for reuse.
