# Supplementary material for: Gut microbiota modulates lung gene expression and metabolism to aid SD rats in adapting to low-pressure hypoxia
Source: Microbiol Spectr. 2025 May 6;13(6):e00045-25. doi: 10.1128/spectrum.00045-25 (PMC12131785; doi:10.1128/spectrum.00045-25)
Supplement: Supplemental material — Fig. S1 to S12. [file spectrum.00045-25-s0001.docx]

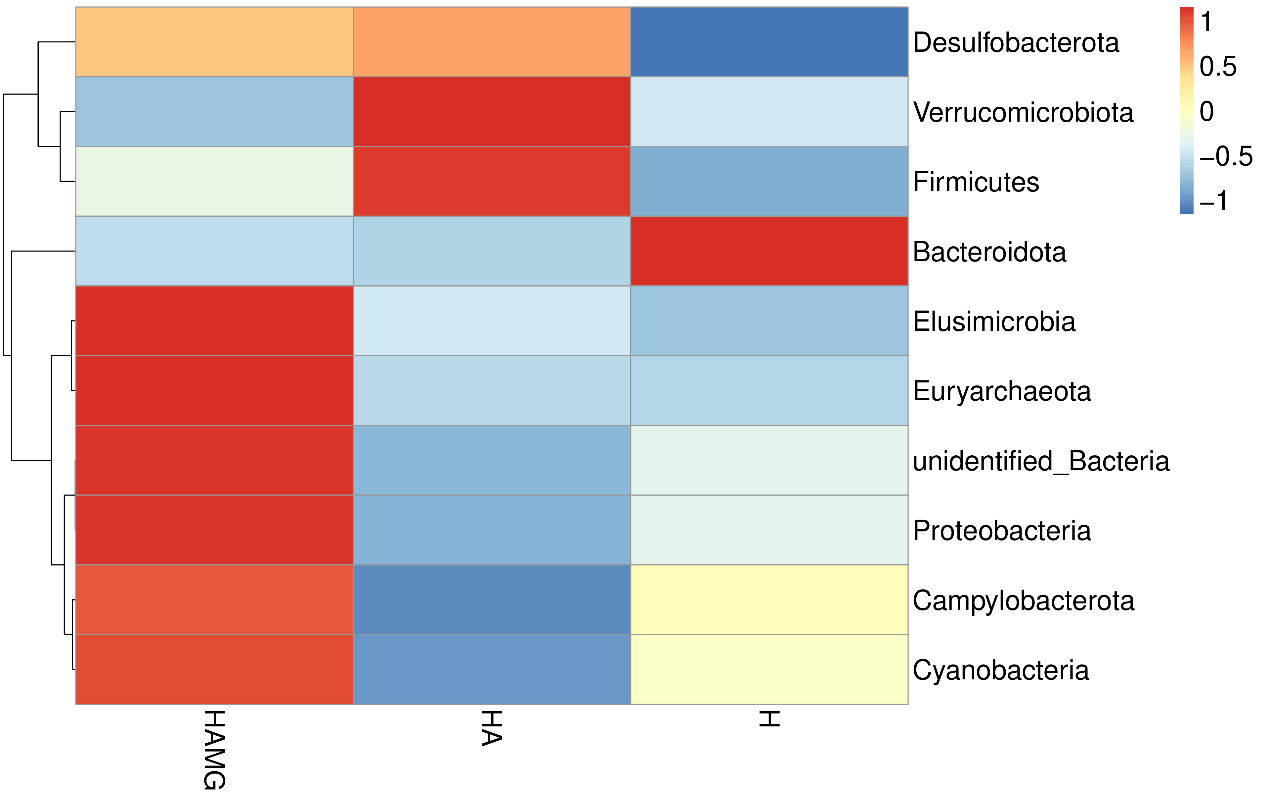


Figure S1. Heatmap of phylum level.


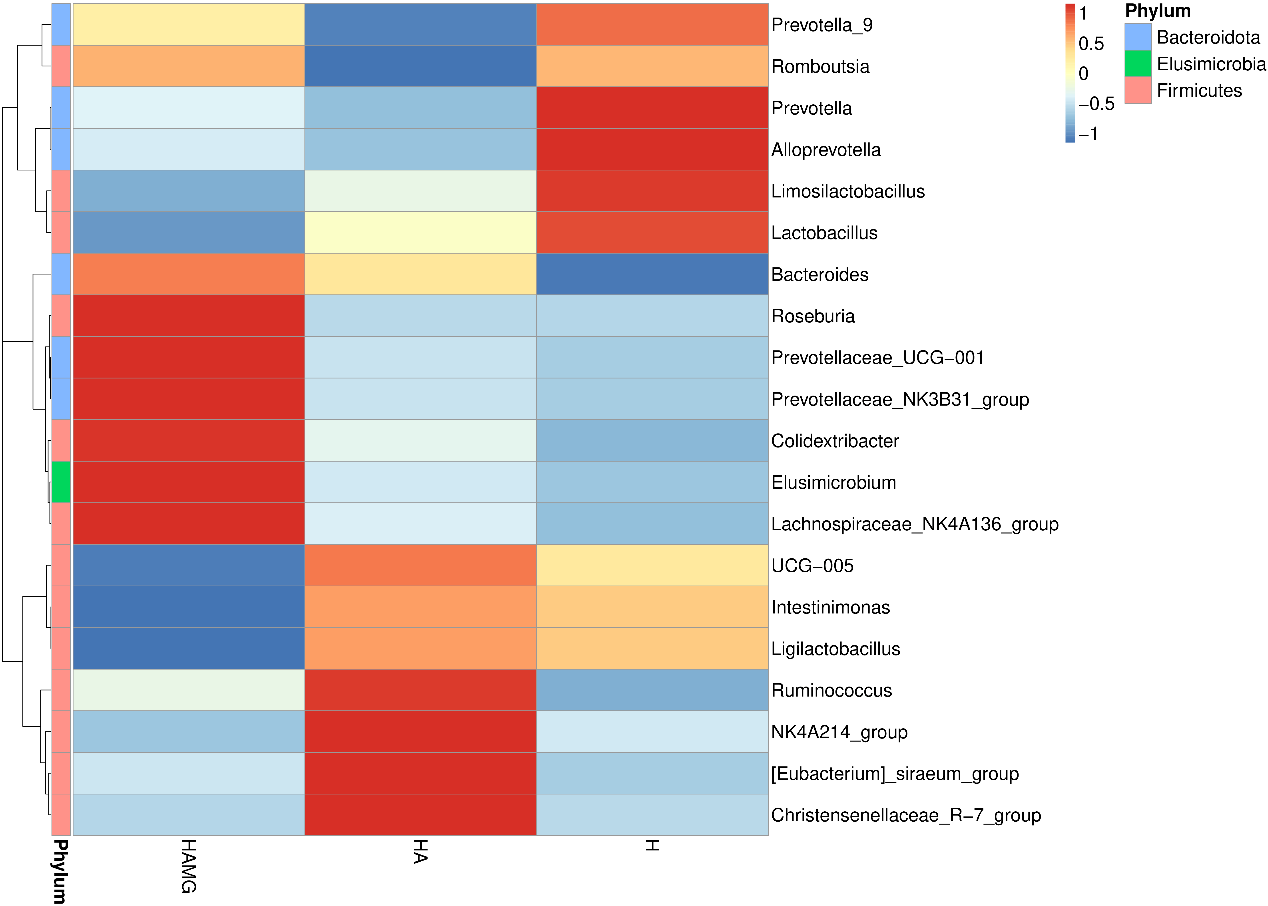


Figure S2. Heatmap of genus level.


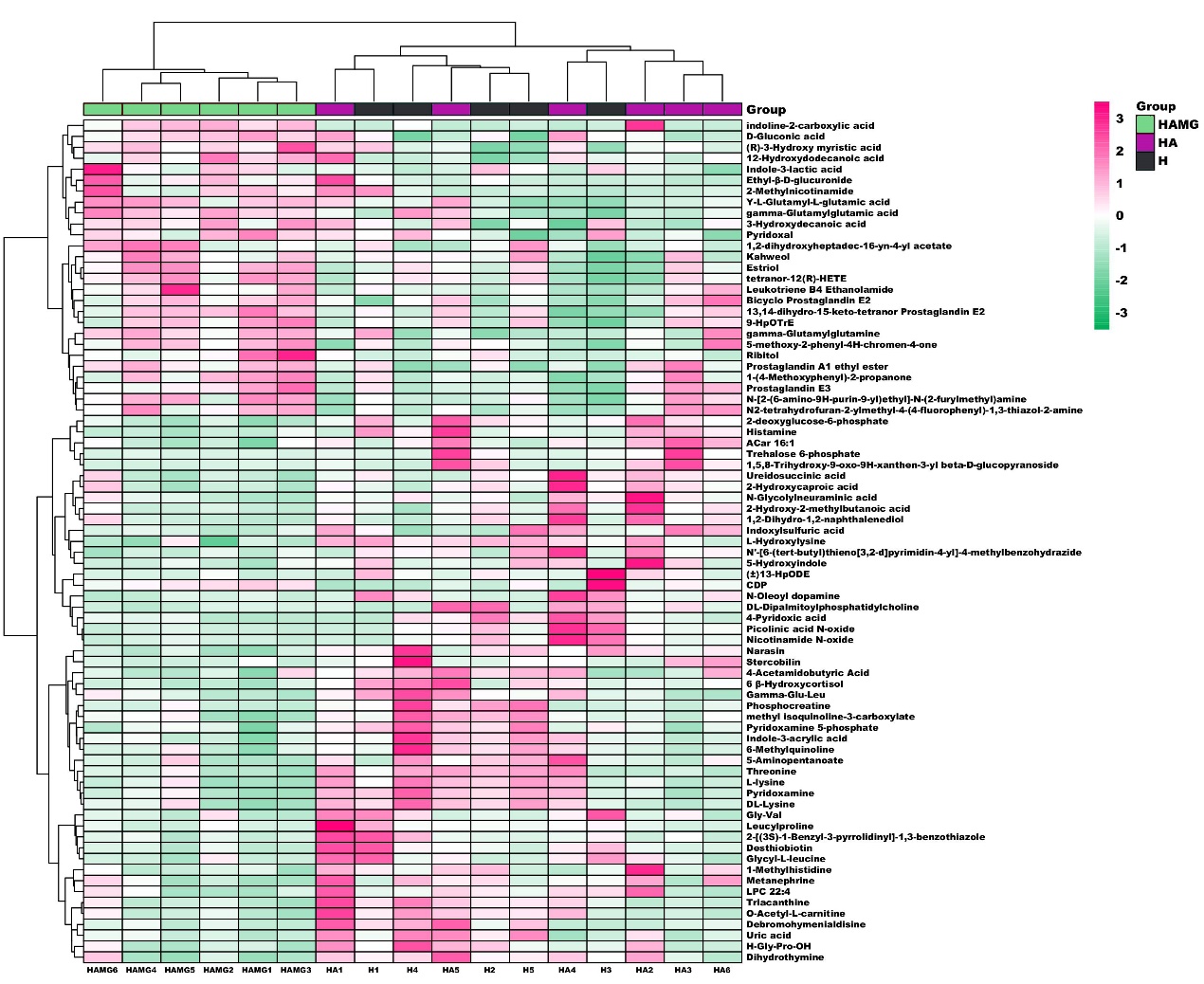


Figure S3. Heatmap of differential metabolites in lung.


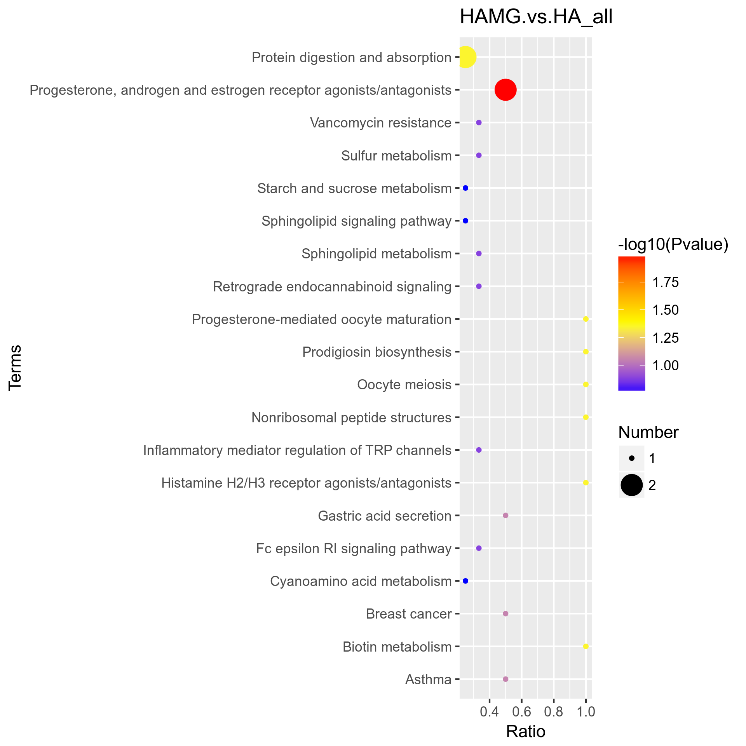


Figure S4. KEGG enrichment pathways in HAMG vs. HA.


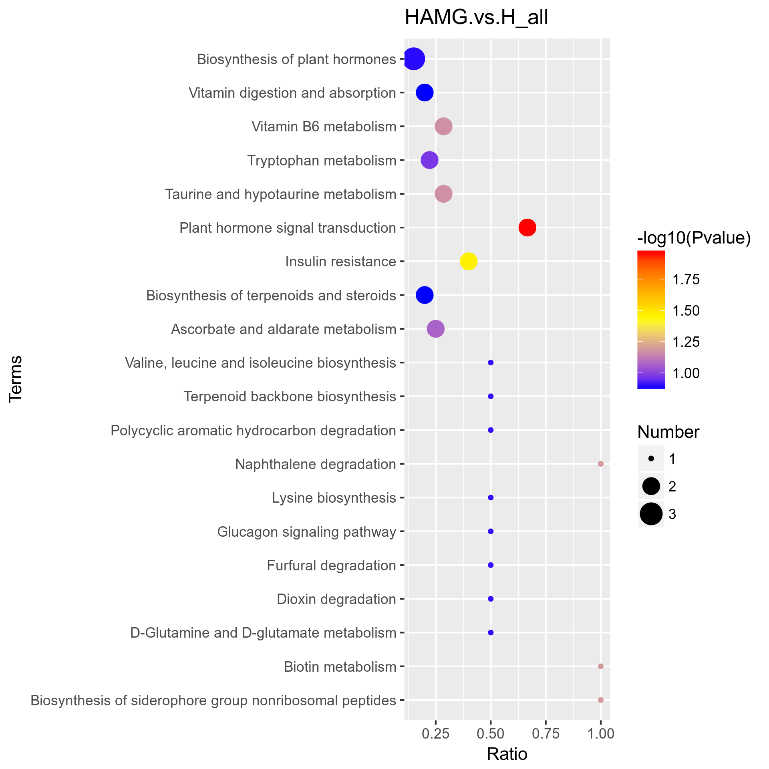


Figure S5. KEGG enrichment pathways in HAMG vs. H.


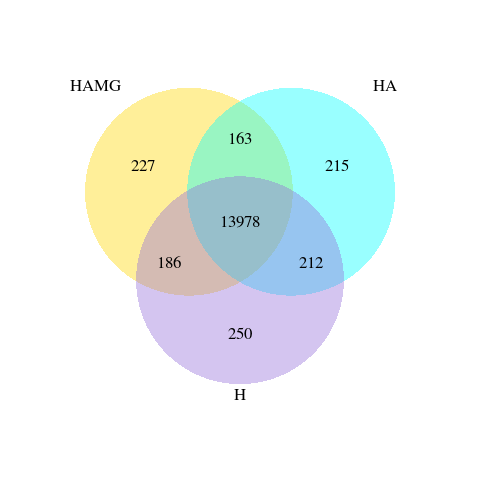


Figure S6. Co-expression Venn diagram of genes.


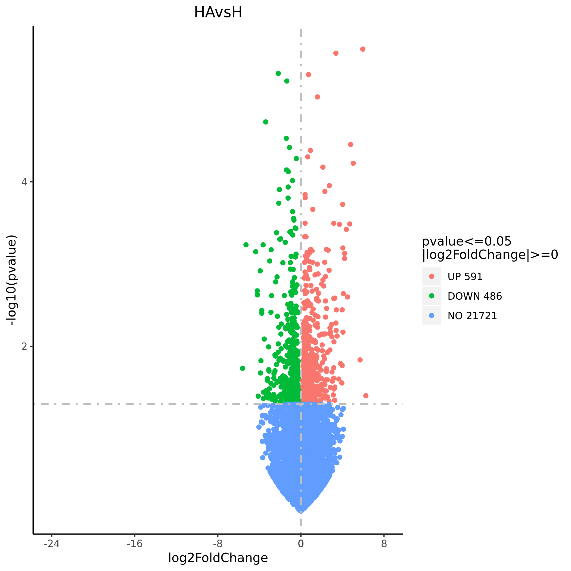


Figure S7. Co-expression Venn diagram of genes.


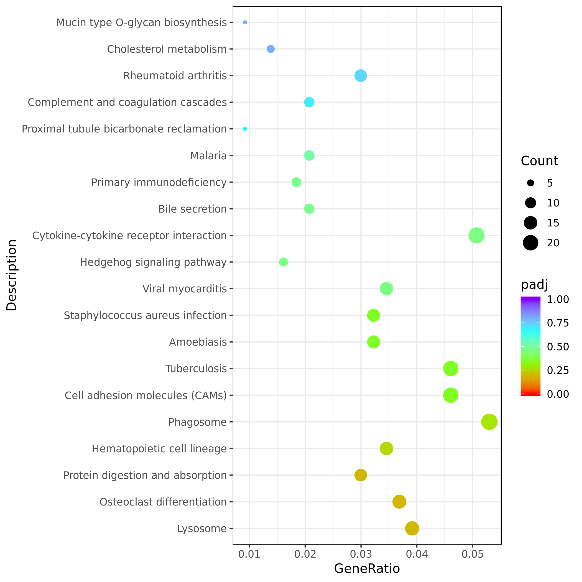


Figure S8. Co-expression Venn diagram of genes.

Supplementary text:

To investigate the impact of microbiota transplantation on the modification of gut microbiota in rats, we compared the alpha diversity of the gut microbiota before and after antibiotic treatment, as well as before and after microbiota transplantation. The results showed significant differences in both the Chao1 and Shannon indices (P < 0.05) (Figure S9). Subsequently, we compared the gut microbiota of the transplantation rats with that of zokors. The PCoA results showed separation and differences between the two groups (Figure S10). Considering the changes in dominant bacteria in the HAMG group compared to the control groups, and the similarities with the dominant bacteria in zokors, such as Lachnospiraceae, Prevotellaceae, and Lachnospiraceae_NK4A136_group, it suggests that the fecal microbiota transplantation from plateau zokors contributed to the reshaping of the gut microbiota (Figure S11,S12).


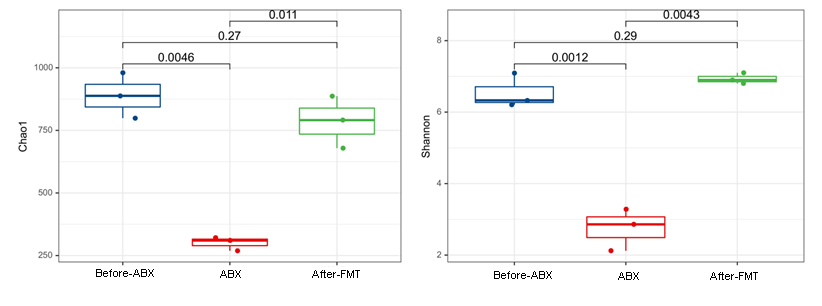


Figure S9. (a) Chao1 index of α-diversity across Before-ABX, ABX, After-FMT. (b) Shannon index of α-diversity across Before-ABX, ABX, After-FMT.


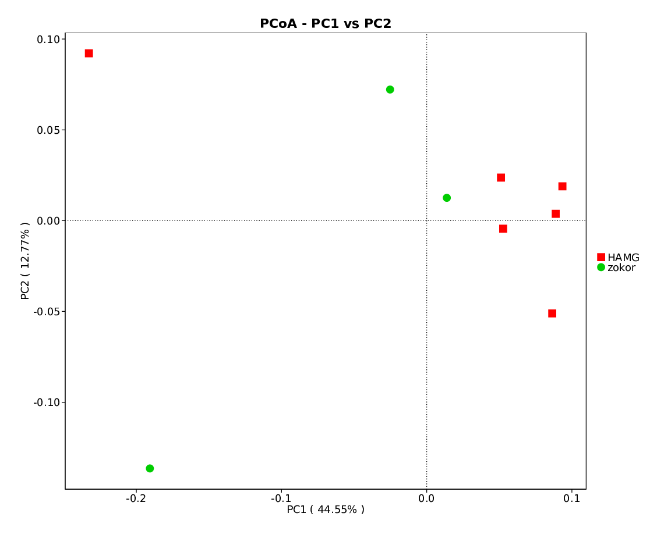


Figure S10. PCoA analysis based on the Unweighted-UniFrac distance.


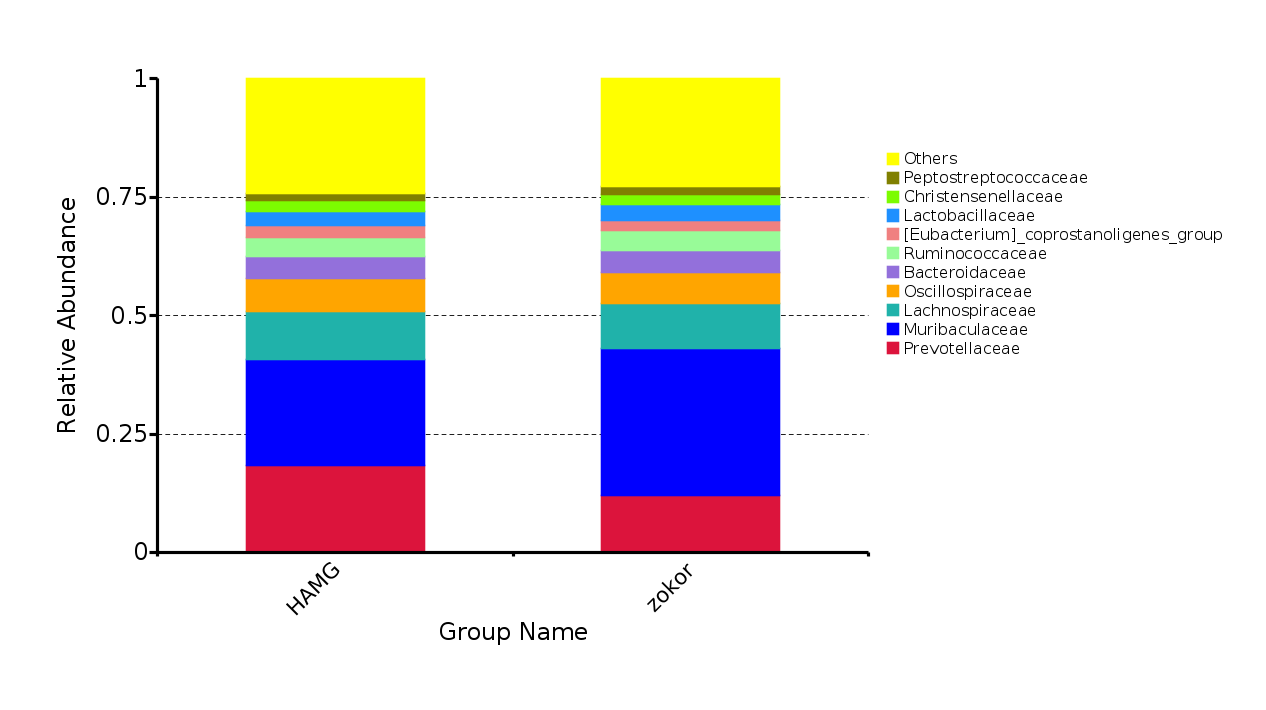


Figure S11. Top 10 family level relative abundance.


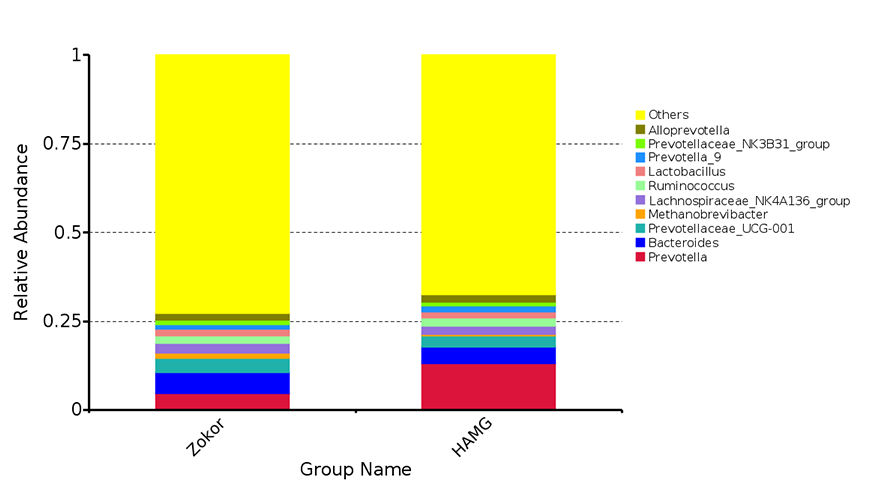


Figure S12. Top 10 genera level relative abundance.
